# From Myotubes to Skeletal Muscle: *Cimicifuga racemosa* Extract Ze 450 Preserves Muscle Mass and Affects Skeletal Muscle Energy Metabolism Under Conditions of Metabolic Stress

**DOI:** 10.3390/cells15191816

**Published:** 2026-10-06

**Authors:** Siyu Chen, Elisabeth Habersatter, Tihomir Kostov, Jürgen Drewe, Georg Boonen, Veronika Butterweck, Patrick Diel

**Affiliations:** 1 Department of Molecular and Cellular Sports Medicine, Institute of Cardiology and Sports Medicine, German Sport University Cologne, Am Sportpark Müngersdorf 6, 50933 Cologne, Germany; siyu.chen@stud.dshs-koeln.de (S.C.); elisabeth.habersatter@zellerag.ch (E.H.); t.kostov@dshs-koeln.de (T.K.); 2 Medical Department, Max Zeller Söhne AG, Seeblickstraße 4, 8590 Romanshorn, Switzerland; juergen.drewe@zellerag.ch (J.D.); georg.boonen@zellerag.ch (G.B.); veronika.butterweck@zellerag.ch (V.B.)

**Keywords:** *Cimicifuga racemosa*, Ze 450, weight loss, skeletal muscle, mitochondrial metabolism, energy, myotubes, exercise mimetic

## Abstract

**Highlights:**

**What are the main findings?**

- *Cimicifuga racemosa* extract Ze 450 failed to induce consistent C2C12 myotube hypertrophy but significantly attenuated dexamethasone-induced myotube atrophy without altering mTOR mRNA expression.
- *Cimicifuga racemosa* extract Ze 450 reduced body weight gain and fat accumulation without decreasing muscle mass or muscle fiber size.

**What are the implications of the main findings?**

- *Cimicifuga racemosa* extract Ze 450 appears to exert anti-atrophic rather than hypertrophic effects on skeletal muscle.
- *Cimicifuga racemosa* extract Ze 450 has the potential to reduce fat mass and body weight while preserving skeletal muscle.

**Abstract:**

Preservation of skeletal muscle is an important consideration when evaluating weight-loss interventions, particularly during menopause, when adverse changes in body composition occur. We investigated the effects of *Cimicifuga racemosa* extract Ze 450 on skeletal muscle. Differentiated C2C12 myotubes were treated with Ze 450, dexamethasone (DEX), or their combination. Non-ovariectomized female Wistar rats were assigned to one of five groups: standard diet, high-fat diet (HF), HF plus voluntary running, HF plus Ze 450 (130 mg/kg/day), or HF plus Ze 450 and voluntary running. Muscle mass and fiber cross-sectional area (CSA) were assessed in the predominantly glycolytic gastrocnemius and oxidative soleus. Markers of mitochondrial content (COX IV and CS), lipid metabolism (ATGL, HSL, FAS, and SREBP-1c), glucose transport (GLUT4), and myogenic signaling (IGF-1) were analyzed. Ze 450 had limited effects on myotube diameter under basal conditions but significantly attenuated DEX-induced myotube atrophy. In vivo, Ze 450 reduced body weight gain and fat accumulation without reducing muscle mass or fiber CSA. Ze 450 differentially affected metabolic markers in the two muscle types, decreasing markers associated with mitochondrial content in the soleus while increasing mitochondrial markers in the gastrocnemius. Taken together, these findings suggest a potential role for Ze 450 in rebalancing skeletal muscle metabolism in a muscle-type-specific manner while preserving skeletal muscle during reductions in body weight gain and adiposity.

## 1. Introduction

During aging, skeletal muscle mass, strength, and regenerative capacity decline progressively, contributing to sarcopenia, a generalized skeletal muscle disorder associated with falls, frailty, functional decline, and premature mortality [1,2]. In the context of obesity treatment, this issue is particularly relevant, as the impact of weight loss depends not only on the magnitude of weight reduction but also on its composition. Skeletal muscle plays a central role in mobility and physical function and contributes to whole-body metabolic homeostasis through insulin-stimulated glucose disposal and broader muscle–organ crosstalk [3]. Therefore, the preservation of skeletal muscle has become an increasingly important consideration in evaluating the overall efficacy of modern weight-loss and anti-obesity therapies [4,5]. Among available strategies, exercise—particularly resistance training—is recognized as an effective approach to preserve skeletal muscle during weight loss [5,6,7]. In contrast, weight loss induced by caloric restriction or pharmacological interventions is often accompanied by reductions in skeletal muscle mass or lean mass [8].

This issue warrants particular attention during the menopausal transition, a period characterized by unfavorable changes in body composition, including increases in fat mass and reductions in lean mass, as well as declines in muscle quality and physical performance [9,10,11,12]. Such changes may prompt attempts at weight loss in midlife women; however, caloric restriction can further exacerbate lean-mass loss when not accompanied by muscle-preserving measures [7,13,14]. With advancing age, a shift from reversible quiescence toward cellular senescence can impair satellite cell self-renewal and regenerative capacity [15]. Satellite cells are resident skeletal muscle stem cells that remain quiescent under homeostatic conditions but become activated following muscle injury to support muscle repair and regeneration [16]. Declining estradiol levels may further impair muscle maintenance by adversely affecting the satellite cell pool and its regenerative capacity [16,17,18].

Despite the role of estradiol in muscle maintenance, evidence for beneficial effects of menopausal hormone therapy on sarcopenia-related outcomes remains inconsistent [19]. Some studies have reported benefits, including a small positive effect on muscle strength [20] and greater gains in muscle mass when estrogen therapy was combined with resistance training in early postmenopausal women [21]. Furthermore, long-term use requires an individualized benefit-risk assessment [22]. At the same time, there is continued interest in nonhormonal treatments with potential relevance to metabolic and skeletal muscle health [3,4,5].

*Cimicifuga racemosa* (L.) Nutt. (black cohosh) extracts, particularly Ze 450, an ethanolic dry extract from the rhizome, are well established for the treatment of menopausal symptoms [23,24,25,26,27]. Detailed phytochemical analyses of *C. racemosa* dry extracts have identified cycloartane-type triterpene glycosides, including actein, 23-epi-26-deoxyactein, cimiracemoside A, and acetylhydroshengmanol and cimigenol glycosides [28]. Importantly, Ze 450 has been shown to act independently of estrogen receptor signaling [29,30], distinguishing it clearly from hormone replacement therapy (HRT). The nonhormonal mode of action and metabolic effects of Ze 450 make it particularly relevant to the investigation of pathways linked to skeletal muscle health.

Mechanistic studies suggest that Ze 450 induces metabolic reprogramming. In HT22 neuronal cells, it suppresses mitochondrial respiration, promotes a shift from oxidative phosphorylation toward glycolysis, and activates AMP-activated protein kinase (AMPK), a central regulator of cellular energy homeostasis [30,31,32]. In C2C12 muscle cells, these effects are accompanied by reduced oxygen consumption rate and increased glucose uptake, supporting the hypothesis that Ze 450 may influence pathways directly relevant to skeletal muscle metabolism [30,31,32]. In vivo, Ze 450 improved glucose metabolism and insulin sensitivity in *ob/ob* mice, while separate observational clinical data in women suggest potentially favorable metabolic associations [33,34]. In addition, Ze 450 has demonstrated beneficial metabolic effects in female rat models, including reduced fat mass gain, lower visceral adiposity, and lower leptin levels, as well as effects observed when combined with voluntary running activity under both physiological and metabolically challenged conditions [35,36].

Although Ze 450 has been shown to reduce adiposity [36], its impact on skeletal muscle remains unclear. Based on the previously observed effects of Ze 450 on adiposity and metabolic regulation, we hypothesized that Ze 450 could influence skeletal muscle responses under metabolic and catabolic challenge. The present study therefore investigated the effects of Ze 450 on skeletal muscle using complementary in vitro and in vivo approaches. In differentiated C2C12 myotubes, the effects on myotube diameter and relevant gene expression were assessed under basal and dexamethasone-induced catabolic stress conditions. In parallel, additional skeletal muscle outcomes were examined in the same experimental cohort of female Wistar rats for which adiposity-related and metabolic findings were previously reported [36]. Specifically, the effects of Ze 450, voluntary running activity, and their combination on skeletal muscle mass and metabolic pathways in *M. gastrocnemius* and *M. soleus* were assessed.

## 2. Materials and Methods

### 2.1. Substances and Stock Solutions for C2C12 Myotube Treatment

Ze 450 (*Cimicifuga racemosa* extract, 60% ethanolic, drug-extract ratio 4.5–8.5:1, batch No. 210301) was provided by Max Zeller Söhne AG (Romanshorn, Switzerland). The extract contained 8% triterpene glycosides. Dihydrotestosterone (DHT; purity ≥ 97.5%) and dexamethasone (DEX; purity ≥ 98%) were obtained from Sigma-Aldrich Chemistry GmbH (Steinheim, Germany). Stock solutions of Ze 450 were prepared at a concentration of 100 mg/mL in 100% DMSO. Stock solutions of DHT and DEX were prepared at a concentration of 2 mM in 100% DMSO. All stock solutions were stored at −20 °C for a maximum of two months. To preserve biological activity, DEX stock solutions were subjected to no more than one freeze–thaw cycle, as repeated freezing may reduce their effectiveness.

### 2.2. Cultivation, Differentiation, and Treatment of C2C12 Cells

Murine C2C12 myoblasts were cultured at 37 °C in a humidified atmosphere containing 5% CO_2_. The growth medium consisted of DMEM supplemented with 20% fetal calf serum (FCS), 4 mM L-glutamine, 1.5 g/L sodium bicarbonate, 1% penicillin/streptomycin, and 1 mM sodium pyruvate. Cells were maintained in T75 culture flasks and passaged approximately twice per week upon reaching 60–70% confluence. The growth medium was replaced 24 h after thawing or passaging and subsequently renewed every two days. For differentiation, cells were seeded into 6-well plates at a density of 1 × 10^4^ cells per well. Upon reaching 90–100% confluence, cells were washed three times with DPBS and switched to differentiation medium consisting of DMEM supplemented with 10% horse serum. The differentiation and growth media were renewed every 24–48 h. Four days after the induction of differentiation, the resulting myotubes were treated with DMSO (vehicle control), DHT (1 µM), DEX (1 µM), Ze 450 (0.1, 1, or 10 µg/mL), or the indicated combinations for 72 h.

### 2.3. C2C12 Myotube Diameter Analysis

Myotube diameter was assessed using an established morphometric approach based on glutaraldehyde-induced autofluorescence, following the methodological principle described by Zheng et al. [37]. In each experiment, two technical replicates per treatment group were prepared. Following the treatment period, the myotubes were washed three times with DPBS, fixed with 2.5% glutaraldehyde (Sigma-Aldrich Chemie GmbH, Steinheim, Germany) diluted in distilled water, and incubated in the dark at 4 °C for 1 h. After fixation, the myotubes were washed three times with DPBS and stored in DPBS at 4 °C until microscopic analysis, which was performed within 8 days of fixation for all treatment groups. Glutaraldehyde-induced autofluorescence enabled visualization of the myotubes using a fluorescence microscope (Axiovert 200 M, Zeiss, Göttingen, Germany). Ten images were acquired from each technical replicate. Images were blinded to treatment group, and the resulting 20 images per treatment were analyzed. From each image, five myotubes were randomly selected, and myotube diameter was measured at four locations along the length of each myotube; the resulting 20 measurements were averaged to yield a single value per image. Measurements were performed using ImageJ software (version 1.52p; NIH, Bethesda, MD, USA).

### 2.4. Animals and Experimental Design

Animal experiments were approved by the local authority (Landesamt für Natur, Umwelt und Verbraucherschutz North Rhine-Westphalia, Germany; protocol no. 81-02.04.2022.A306), and a non-technical project summary was registered (BfR, Germany; NTP-ID: 00054883-1-1).

The study design was described in detail previously [36]. Briefly, 30 non-ovariectomized female Wistar rats were subjected to a 12-week intervention and randomly assigned to five groups (*n* = 6 per group): standard diet (SD), high-fat diet (HF), HF + activity (A), HF + Ze 450, and HF + Ze 450 + A.

Animals had ad libitum access to food and water and were housed under standard conditions. For the designated groups, voluntary running activity was enabled by providing running wheels overnight, and running distances were recorded daily. Body weight was monitored daily, and food intake was recorded per cage. After 12 weeks, animals were fasted overnight and euthanized for tissue collection. Skeletal muscle samples (*M. gastrocnemius* and *M. soleus*) were collected for subsequent analyses.

### 2.5. Diet and Ze 450 Administration

Animals received either a standard phytoestrogen-free chow or a high-fat diet as described previously [36]. Ze 450 (*Cimicifuga racemosa* extract, 60% ethanolic, drug-extract ratio 4.5–8.5:1, batch No. 210301) was incorporated into the high-fat diet to achieve an intended daily dose of 130 mg/kg body weight, as previously established [36]. Because Ze 450 was administered via the diet and food intake was recorded per cage, individual Ze 450 intake could not be determined. Successful incorporation and stability of Ze 450 in the diet were confirmed by UPLC-MS analysis of triterpene glycosides, as described previously [36]. Food intake was recorded per cage, and metabolizable energy intake was calculated based on manufacturer specifications.

### 2.6. Skeletal Muscle Dissection and Weighing

Following euthanasia, the hindlimb musculature was rapidly exposed, and the gastrocnemius and soleus muscles were dissected under sterile conditions. The overlying connective tissue and fascia were carefully removed to minimize damage to muscle fibers and preserve overall structural integrity.

The distal tendon (Achilles tendon) was first transected, and the gastrocnemius–soleus complex was gently detached from surrounding tissues while the proximal tendinous attachments were kept intact, thereby maintaining continuity from proximal to distal ends and facilitating subsequent separation.

The gastrocnemius and soleus muscles were then separated along the natural intermuscular plane (intermuscular septum) using blunt dissection to minimize mechanical disruption to adjacent tissues. After complete separation, the proximal tendons were transected, and the intact muscles were excised.

Muscle samples obtained using this procedure were free of visible non-muscle tissue contamination. Surface blood was gently removed, after which the muscles were weighed and their weights were recorded. The samples were subsequently divided into two portions: one portion was embedded in OCT compound and snap-frozen in liquid nitrogen for morphological analysis (cryosectioning), whereas the other was snap-frozen in liquid nitrogen for molecular analyses.

### 2.7. H&E Staining and Muscle Fiber Cross-Sectional Area

Muscle fiber cross-sectional area (CSA) was assessed on transverse cryosections of skeletal muscle. H&E staining of the cryosections was performed as previously described [38].

For each animal, at least five transverse sections were obtained. Sections meeting predefined quality criteria (i.e., intact morphology without folding or tearing) were selected for analysis. Images were acquired at 20× magnification, and at least ten fields of view were captured per section using a systematic zigzag sampling approach to avoid overlapping regions.

Individual muscle fibers were identified and outlined using ImageJ software (version 1.54f, National Institutes of Health, Bethesda, MD, USA) to determine CSA. Only fibers with intact and clearly defined borders were included in the analysis, whereas distorted or obliquely sectioned fibers were excluded. CSA was determined by manual delineation of clearly defined muscle fiber boundaries. Illumination intensity and staining color were not used as quantitative variables.

A minimum of 200 muscle fibers per animal were analyzed (at least 20 fibers per image). Previous studies have demonstrated that the variability of CSA estimates markedly decreases and stabilizes when at least 150 fibers are measured [39]. The number of fibers analyzed in the present study exceeded this threshold to improve measurement accuracy and representativeness.

For presentation, color saturation was reduced and color balance was adjusted across each representative histological image, without local retouching or changes to image geometry. These adjustments were applied solely for figure presentation and did not affect CSA quantification.

### 2.8. Intramuscular Triglyceride (IMTG) Measurement

Intramuscular triglyceride (IMTG) content was determined using a commercial triglyceride assay kit (ab65336, Abcam, Cambridge, UK) according to the manufacturer’s instructions. Within each group, muscle samples were pooled into a single sample, homogenized, and centrifuged to obtain supernatants for analysis as instructed. Absorbance was measured at 570 nm in 96-well plates using a microplate reader. Triglyceride concentrations were calculated based on a standard curve and normalized to protein concentration.

### 2.9. Western Blotting

For skeletal muscle tissue, samples were homogenized in lysis buffer containing protease and phosphatase inhibitors, and protein extracts were collected after centrifugation. Protein concentration was determined using a DC Protein Assay. Proteins were separated by SDS-PAGE, transferred to membranes, and incubated with antibodies against COX IV (4850S, Cell Signaling Technology, Davers, MA, USA), citrate synthase (14309S, Cell Signaling Technology, USA), FAS (AB22759, Abcam, Cambridge, UK), and β-actin (MAB1501R, Merck, Darmstadt, Germany). When required, membranes were stripped using stripping buffer (21059, Thermo Fisher Scientific, Waltham, MA, USA) and reprobed. Protein expression was normalized to β-actin. Western blotting was performed as previously described [40,41].

### 2.10. Real-Time PCR

For C2C12 myotubes, total RNA was isolated using TRIzol^®^ (Invitrogen, Carlsbad, CA, USA) reagent following the treatment period. RNA concentration and purity were determined spectrophotometrically, and integrity was verified by agarose gel electrophoresis. cDNA synthesis was performed using the QuantiTect^®^ Reverse Transcription Kit (Qiagen, Hilden, Germany) according to the manufacturer’s instructions. Quantitative real-time PCR (RT-qPCR) was carried out using SYBR Green chemistry. Gene expression levels of IGF-1 and mTOR were analyzed in three independent experiments. Relative gene expression was calculated using the ΔΔCt method (2^−ΔΔCt^), with normalization to a housekeeping gene. Primer sequences are listed in Table 1. For skeletal muscle tissue, total RNA was extracted using TRIzol^®^ reagent. RNA processing, cDNA synthesis, and RT-qPCR were performed as described above. Relative mRNA expression of target genes was calculated after normalization to a housekeeping gene. RT-qPCR was performed as previously described [41].

### 2.11. Statistical Analysis

**Figure A1:**
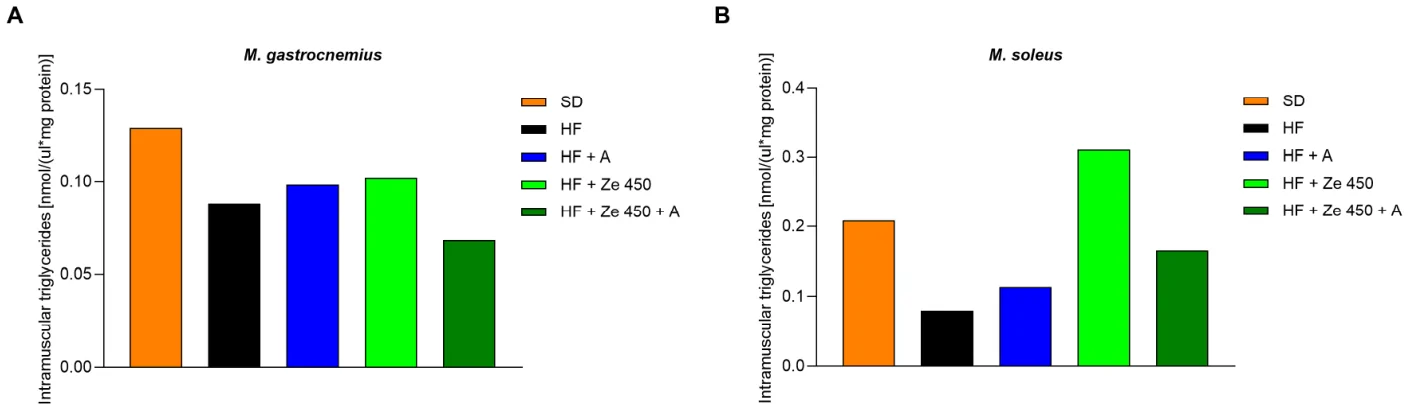
Exploratory measurement of intramuscular triglyceride (IMTG) content in skeletal muscle of high-fat diet-fed rats. IMTG content was determined in (**A**) *M. gastrocnemius* and (**B**) *M. soleus* from animals fed a standard diet (SD), a high-fat diet (HF), HF plus voluntary physical activity (HF + A), HF plus Ze 450 (130 mg/kg body weight/day), or HF plus Ze 450 and voluntary physical activity (HF + Ze 450 + A). For each experimental group, muscle tissue from six animals was combined into a single pooled sample, which was measured once. Consequently, the values represent single descriptive measurements. Measures of variability and inferential statistical analyses are therefore not applicable. The data are preliminary and hypothesis-generating and do not establish group or treatment effects.

Statistical analysis was performed using GraphPad Prism version 10 (GraphPad Software, Boston, MA, USA). Data are presented as mean ± standard deviation (SD), unless otherwise stated. Data from independent in vitro experiments were analyzed using repeated-measures one-way ANOVA, with the experimental runs treated as matched observations. Where applicable, the Geisser–Greenhouse correction was applied. Ordinary one-way ANOVA was used for comparisons among independent groups in the in vivo experiments. ANOVA was followed by Dunnett’s multiple-comparisons test for comparisons with a single reference group or by Šídák’s multiple-comparisons test for prespecified comparisons involving different reference groups, as specified in the figure legends. Reported *p* values from multiple comparisons were adjusted for multiplicity. The significance level was set at α = 0.05. Formal tests of normality and homogeneity of variance were not performed because of the limited sample sizes and the low statistical power of such tests under these conditions. The exploratory IMTG data shown in Figure A1 were excluded from inferential statistical analysis because only one pooled measurement was available per muscle and treatment group.

## 3. Results

### 3.1. Effects of Ze 450 on C2C12 Myotube Diameter

**Figure 1:**
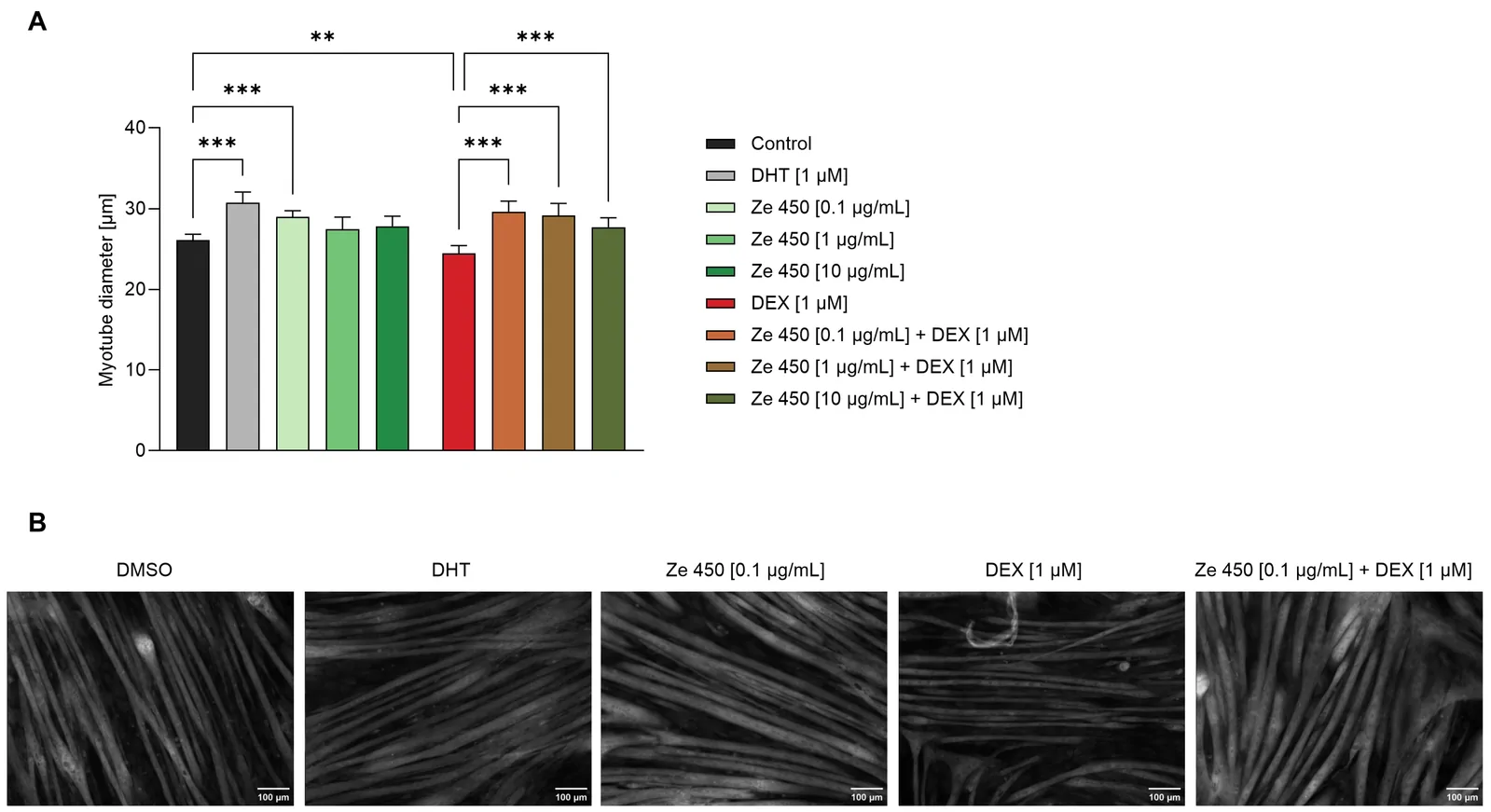
Effects of Ze 450 on C2C12 myotube diameter. (**A**) Myotube diameter (µm) following treatment with 1 µM DHT, Ze 450 (0.1, 1, and 10 µg/mL), 1 µM DEX, or DEX in combination with Ze 450. (**B**) Representative images of C2C12 myotubes. Data are presented as mean ± standard deviation from *n* = 3 independent experiments; multiple myotubes were measured per condition in each experiment. Statistical significance was assessed using repeated-measures one-way ANOVA with the Geisser–Greenhouse correction, followed by Šídák’s multiple-comparisons test. ** *p* < 0.01, *** *p* < 0.001.

As shown in Figure 1, DHT treatment significantly increased myotube diameter by 17% compared with the vehicle control. Ze 450 treatment resulted in a small increase in myotube diameter but only at the lowest Ze 450 concentration.

In contrast, cotreatment with Ze 450 significantly attenuated the DEX-induced reduction in myotube diameter at all tested Ze 450 concentrations.

### 3.2. Effects of Ze 450 on IGF-1 and mTOR mRNA Expression in C2C12 Myotubes

**Figure 2:**
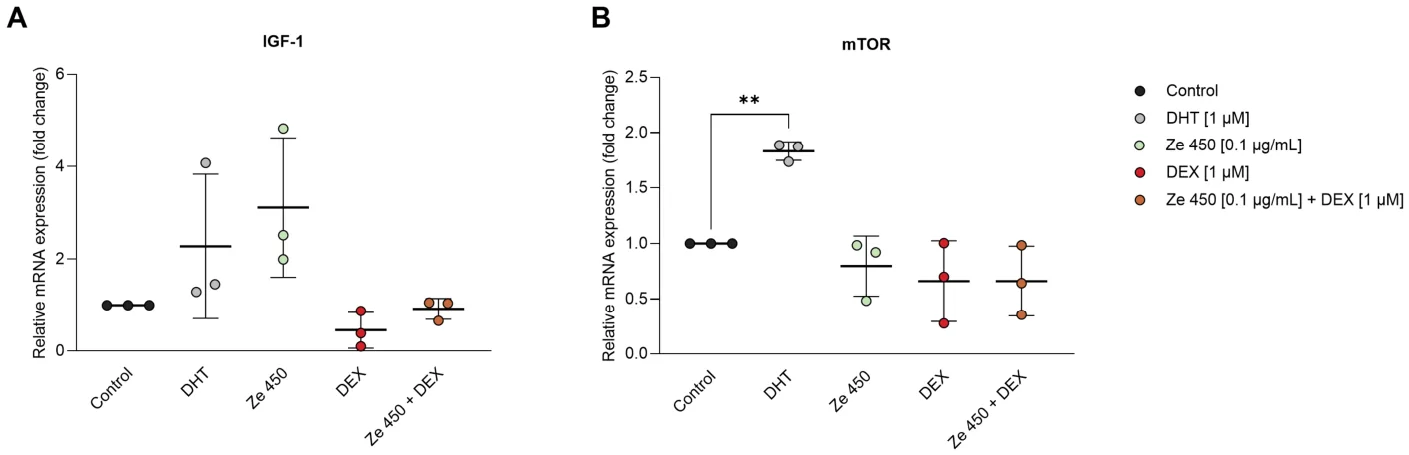
Effects of Ze 450 on IGF-1 and mTOR mRNA expression in C2C12 myotubes. (**A**) Relative IGF-1 mRNA expression levels in C2C12 myotubes following treatment with DHT (1 µM), Ze 450 (0.1 µg/mL), DEX (1 µM), or DEX + Ze 450. (**B**) Relative mTOR mRNA expression levels under the same treatment conditions. Data are presented as mean ± standard deviation of *n* = 3 independent experiments. Statistical analysis was performed using repeated-measures one-way ANOVA followed by Dunnett’s multiple-comparisons test. ** *p* < 0.01.

As shown in Figure 2A, DHT (1 μM) and Ze 450 (0.1 µg/mL) showed nonsignificant trends toward increased IGF-1 mRNA expression, whereas DEX (1 μM) showed numerically lower IGF-1 mRNA expression than the vehicle control. Cotreatment with Ze 450 showed a nonsignificant trend toward attenuating this numerical decrease in IGF-1 mRNA expression observed with DEX. DHT significantly increased mTOR mRNA expression, whereas Ze 450, DEX, and the combination of DEX and Ze 450 did not significantly affect mTOR mRNA expression (Figure 2B).

### 3.3. Effects of Ze 450 on Body Weight and Skeletal Muscle Mass of High-Fat Diet-Fed Rats

**Figure 3:**
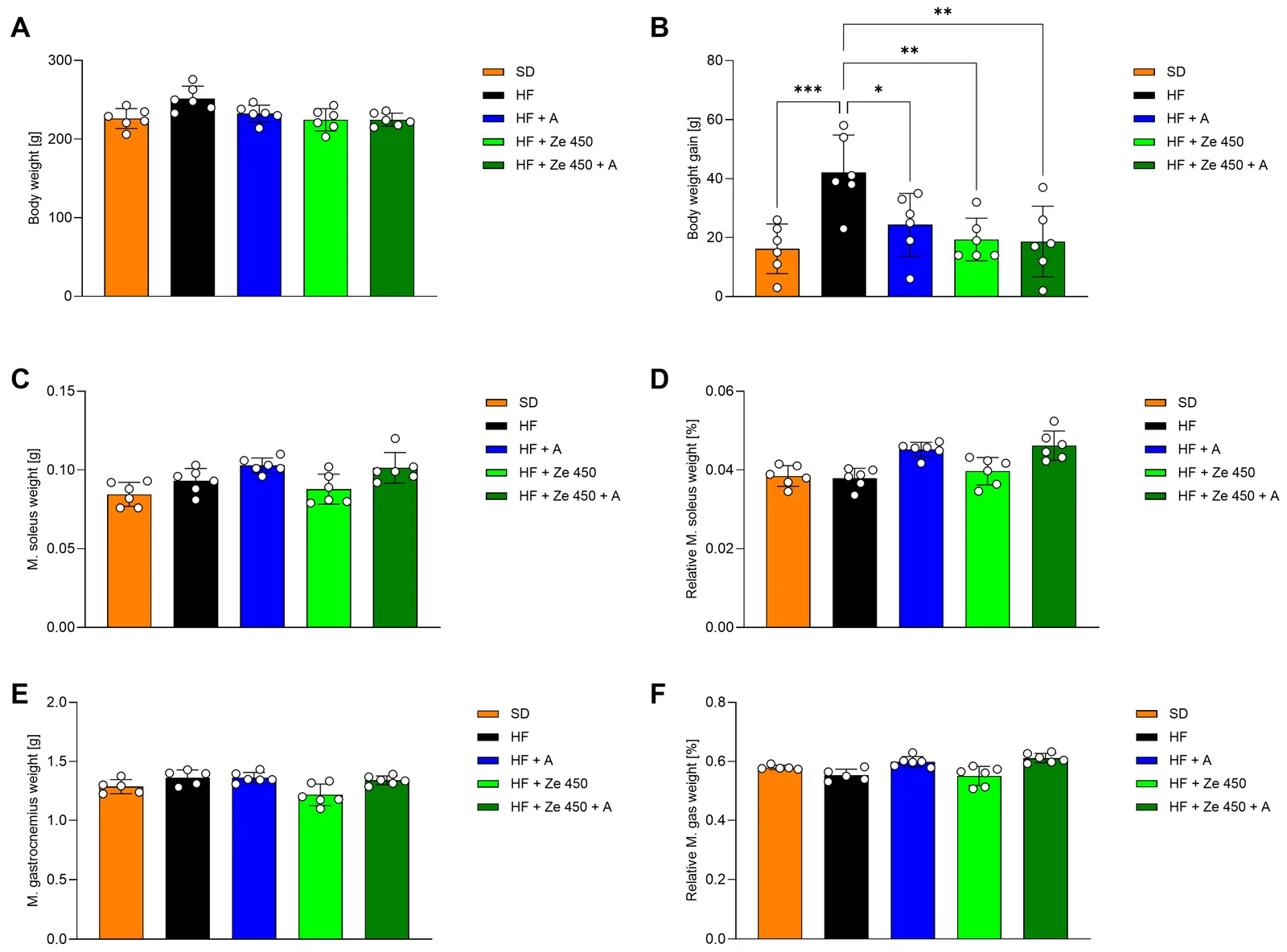
Effects of Ze 450 on body weight and skeletal muscle mass of high-fat diet-fed rats. (**A**) Body weight and (**B**) body weight gain at the study endpoint in animals fed a standard diet (SD), high-fat diet (HF), HF + A (= voluntary running activity), HF + Ze 450 (130 mg/kg body weight/day), or HF + Ze 450 + A. (**C**,**D**) Absolute and body weight-normalized mass, respectively, of *M. soleus*. (**E**,**F**) Absolute and body weight-normalized mass, respectively, of *M. gastrocnemius*. All data are presented as mean ± standard deviation (6 animals per group). Statistical analysis was performed using one-way ANOVA followed by Dunnett’s multiple-comparisons test, comparing SD and treatment groups to the HF control. * *p* < 0.05, ** *p* < 0.01, *** *p* < 0.001.

As shown in Figure 3A,B, the HF diet significantly increased body weight gain compared with the standard diet. This increase was attenuated by Ze 450 (130 mg/kg body weight/day), voluntary running activity, and their combination. These body weight findings from the same cohort have been reported previously [36]. Voluntary running activity showed a nonsignificant trend toward greater gastrocnemius mass. Despite reducing body weight gain, Ze 450 did not significantly affect soleus or gastrocnemius mass (Figure 3C–F).

### 3.4. Effects of Ze 450 on Skeletal Muscle Fiber Cross-Sectional Area (CSA) of High-Fat Diet-Fed Rats

**Figure 4:**
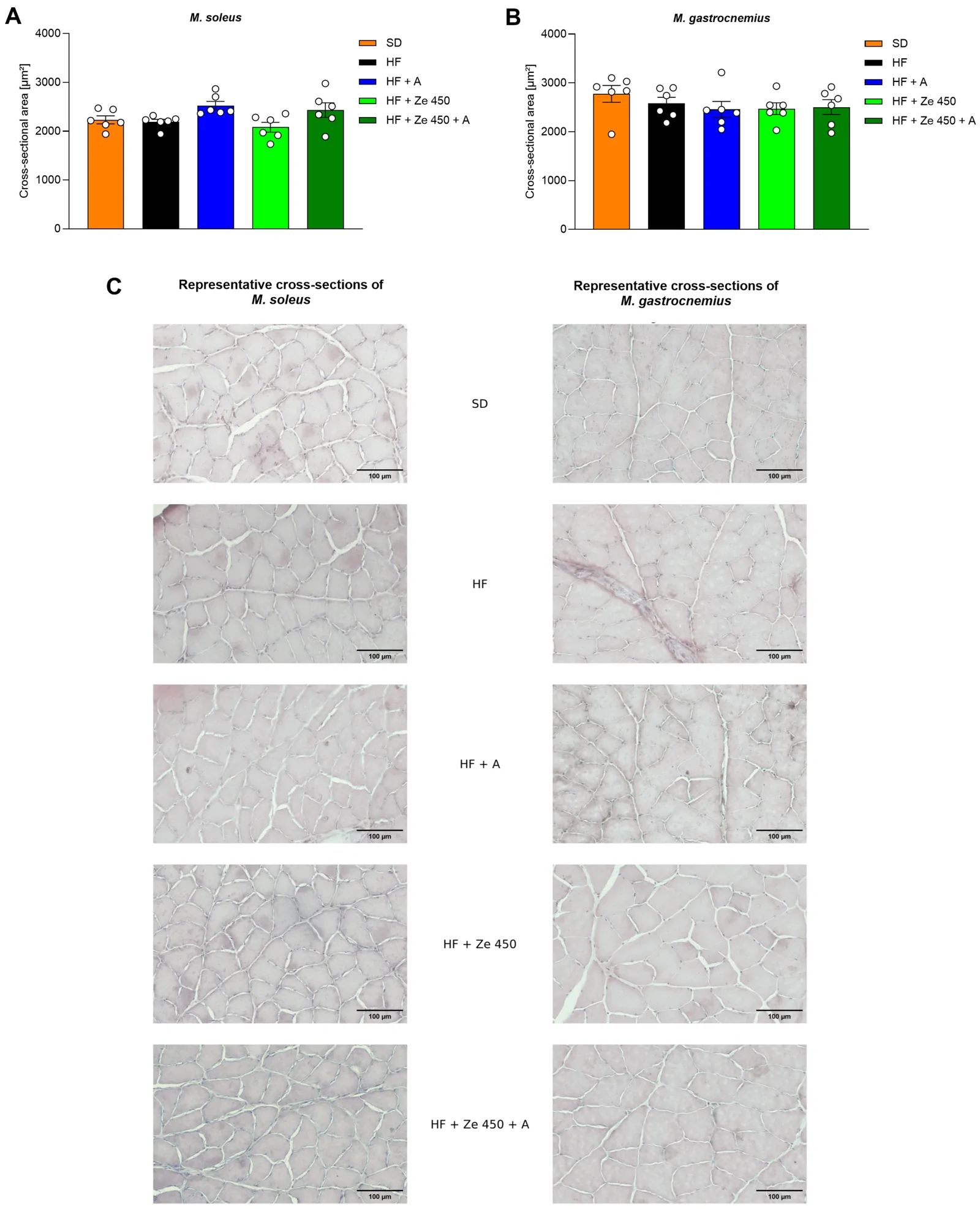
Effects of Ze 450 on skeletal muscle fiber cross-sectional area (CSA) of high-fat diet-fed rats. (**A**,**B**) Skeletal muscle fiber cross-sectional area in *M. soleus* and *M. gastrocnemius* from animals fed a standard diet (SD), high-fat diet (HF), HF + A (= voluntary running activity), HF + Ze 450 (130 mg/kg body weight/day), or HF + Ze 450 + A. (**C**) Representative images of *M. soleus* and *M. gastrocnemius*. All data are presented as mean ± standard deviation (6 animals per group). Statistical analysis was performed using one-way ANOVA followed by Dunnett’s multiple-comparisons test, comparing SD and treatment groups to the HF control.

As shown in Figure 4A, voluntary running activity produced a nonsignificant trend toward increased soleus muscle fiber CSA. No statistically significant differences in soleus or gastrocnemius muscle fiber CSA were observed among the other treatment groups.

### 3.5. Effects of Ze 450 on Mitochondrial Content-Associated Markers in Skeletal Muscle of High-Fat Diet-Fed Rats

**Figure 5:**
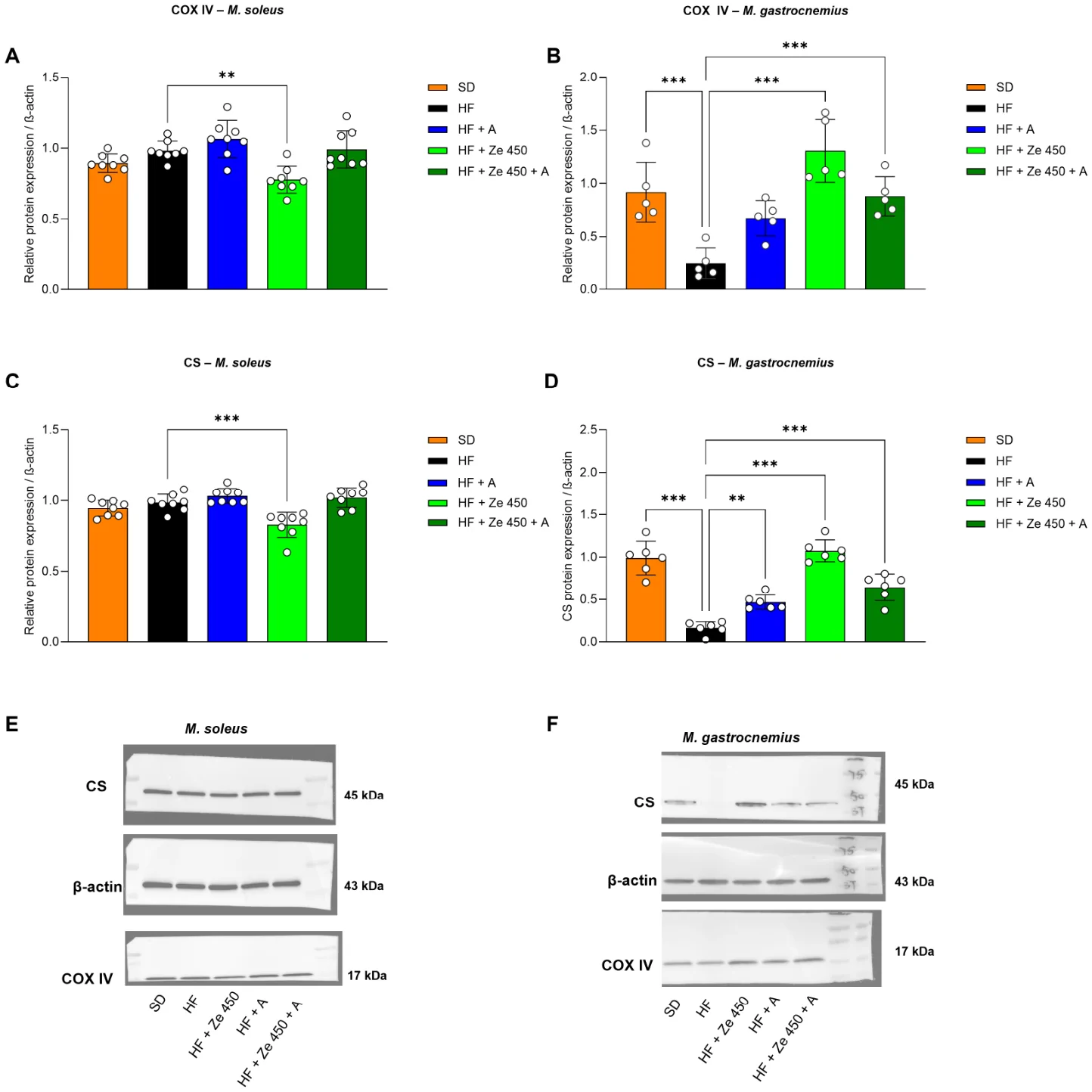
Effects of Ze 450 on mitochondrial metabolic biomarkers in skeletal muscle of high-fat diet-fed rats. (**A**,**C**) Relative protein expression of CS and COX IV normalized to β-actin in *M. soleus* and (**B**,**D**) *M. gastrocnemius* from animals fed a standard diet (SD), high-fat diet (HF), HF + A (=voluntary running activity), HF + Ze 450 (130 mg/kg body weight/day), or HF + Ze 450 + A. (**E**,**F**) Representative Western blot images. All data are presented as mean ± standard deviation from pooled samples (6 animals per group). At least five technical replicates were performed for each experiment. Statistical analysis was performed using one-way ANOVA followed by Dunnett’s multiple-comparisons test, comparing SD and treatment groups to the HF control. ** *p* < 0.01, *** *p* < 0.001.

The expression levels of cytochrome c oxidase and citrate synthase correlate with mitochondrial content [42]. As shown in Figure 5, cytochrome c oxidase subunit IV (COX IV) and citrate synthase (CS) protein levels showed similar patterns within each muscle. In the soleus, Ze 450 reduced the expression of both proteins, whereas concomitant voluntary running activity attenuated these reductions. In the gastrocnemius, the HF diet reduced COX IV and CS protein levels, whereas voluntary running activity, Ze 450, and their combination attenuated these reductions.

### 3.6. Effects of Ze 450 on Lipolysis-Associated Gene Expression in Skeletal Muscle of High-Fat Diet-Fed Rats

**Figure 6:**
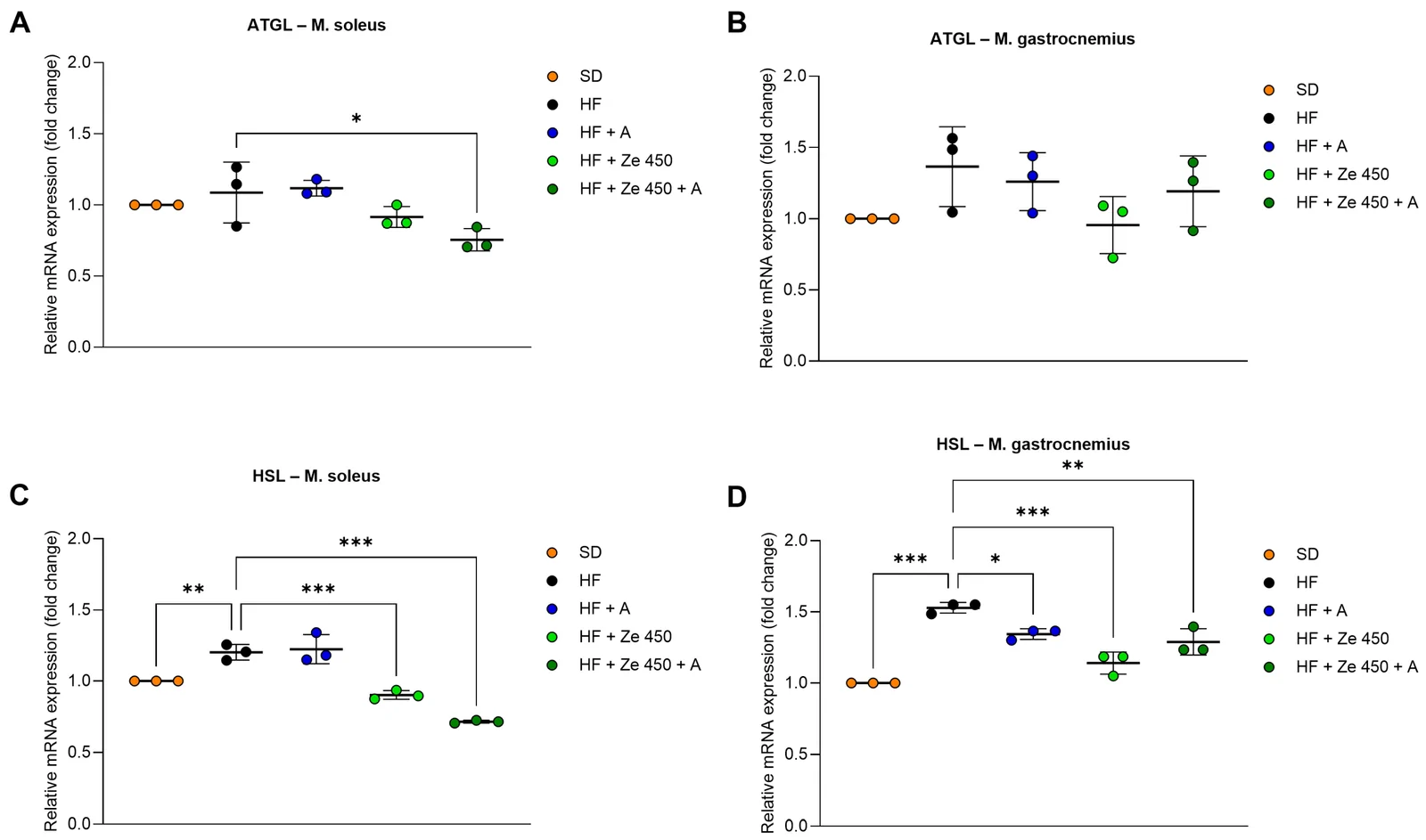
Effects of Ze 450 on lipolysis-associated gene expression in skeletal muscle of high-fat diet-fed rats. (**A**,**C**) Relative mRNA expression of *Pnpla2* (ATGL) and *Lipe* (HSL) normalized to *mt-Co1* (COX1) in *M. soleus* and (**B**,**D**) *M. gastrocnemius* from animals fed a standard diet (SD), high-fat diet (HF), HF + A (=voluntary running activity), HF + Ze 450 (130 mg/kg body weight/day), or HF + Ze 450 + A. All data are presented as mean ± standard deviation from *n* = 3 experiments. Samples were pooled from 6 animals per group prior to analysis. Statistical analysis was performed using one-way ANOVA followed by Dunnett’s multiple-comparisons test, comparing SD and treatment groups to the HF control. * *p* < 0.05, ** *p* < 0.01, *** *p* < 0.001.

As shown in Figure 6, the mRNA expression levels of adipose triglyceride lipase (ATGL) and hormone-sensitive lipase (HSL) exhibited similar patterns within each muscle type, although not all comparisons reached statistical significance.

In the soleus, the high-fat diet significantly increased HSL mRNA expression, whereas ATGL mRNA expression was numerically higher than in the SD group. Ze 450 alone and in combination with voluntary running activity significantly reduced HSL mRNA expression compared with the HF group. ATGL mRNA expression was significantly lower only in the combination group, while Ze 450 alone produced a numerical reduction. Running activity alone did not significantly affect either marker.

In the gastrocnemius, the HF diet significantly increased HSL mRNA expression, whereas the increase in ATGL mRNA expression was not statistically significant. Ze 450, voluntary running activity, and their combination significantly reduced HSL mRNA expression compared with the HF group. ATGL mRNA expression was numerically lower with Ze 450 alone, but no statistically significant differences were detected in the comparisons with the HF group.

### 3.7. Effects of Ze 450 on Lipogenesis-Associated Biomarkers in Skeletal Muscle of High-Fat Diet-Fed Rats

**Figure 7:**
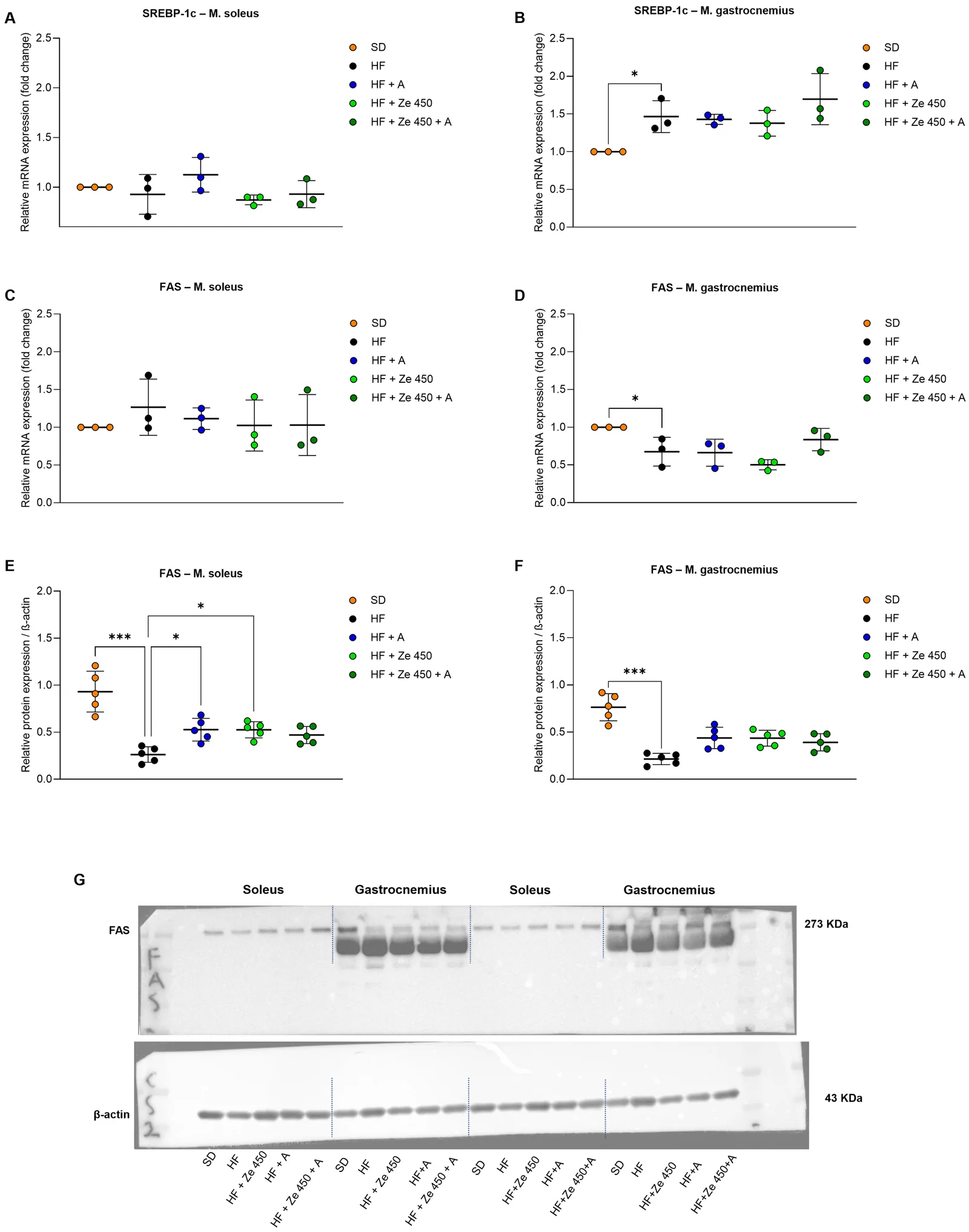
Effects of Ze 450 on lipogenesis-associated biomarkers in skeletal muscle of high-fat diet-fed rats. (**A**,**C**,**E**) Relative mRNA expression of *Srebf1* (SREBP-1c) and *Fasn* (FAS) normalized to *mt-Co1* (COX1) and FAS protein expression level in *M. soleus* and (**B**,**D**,**F**) *M. gastrocnemius* from animals fed a standard diet (SD), high-fat diet (HF), HF + A (=voluntary running activity), HF + Ze 450 (130 mg/kg body weight/day), or HF + Ze 450 + A. (**G**) Representative Western blot images. All data are presented as mean ± standard deviation from at least *n* = 3 experiments. Samples were pooled from 6 animals per group prior to analysis. Statistical analysis was performed using one-way ANOVA followed by Dunnett’s multiple-comparisons test, comparing SD and treatment groups to the HF control. * *p* < 0.05, *** *p* < 0.001.

As shown in Figure 7, the changes in lipogenesis-associated mRNA expression were more pronounced in the gastrocnemius muscle, where the high-fat diet increased SREBP-1c mRNA and decreased FAS mRNA expression. FAS protein expression was downregulated by the high-fat diet in *M. soleus* and *M. gastrocnemius*. In the soleus, voluntary running activity and Ze 450 alone significantly increased FAS protein expression compared with the HF group, whereas their combination produced a numerical increase. In the gastrocnemius, FAS protein expression was numerically higher in all intervention groups than in the HF group, but these differences did not reach statistical significance.

### 3.8. Effects of Ze 450 on Glucose Transport and Myogenic Gene Expression in Skeletal Muscle of High-Fat Diet-Fed Rats

**Figure 8:**
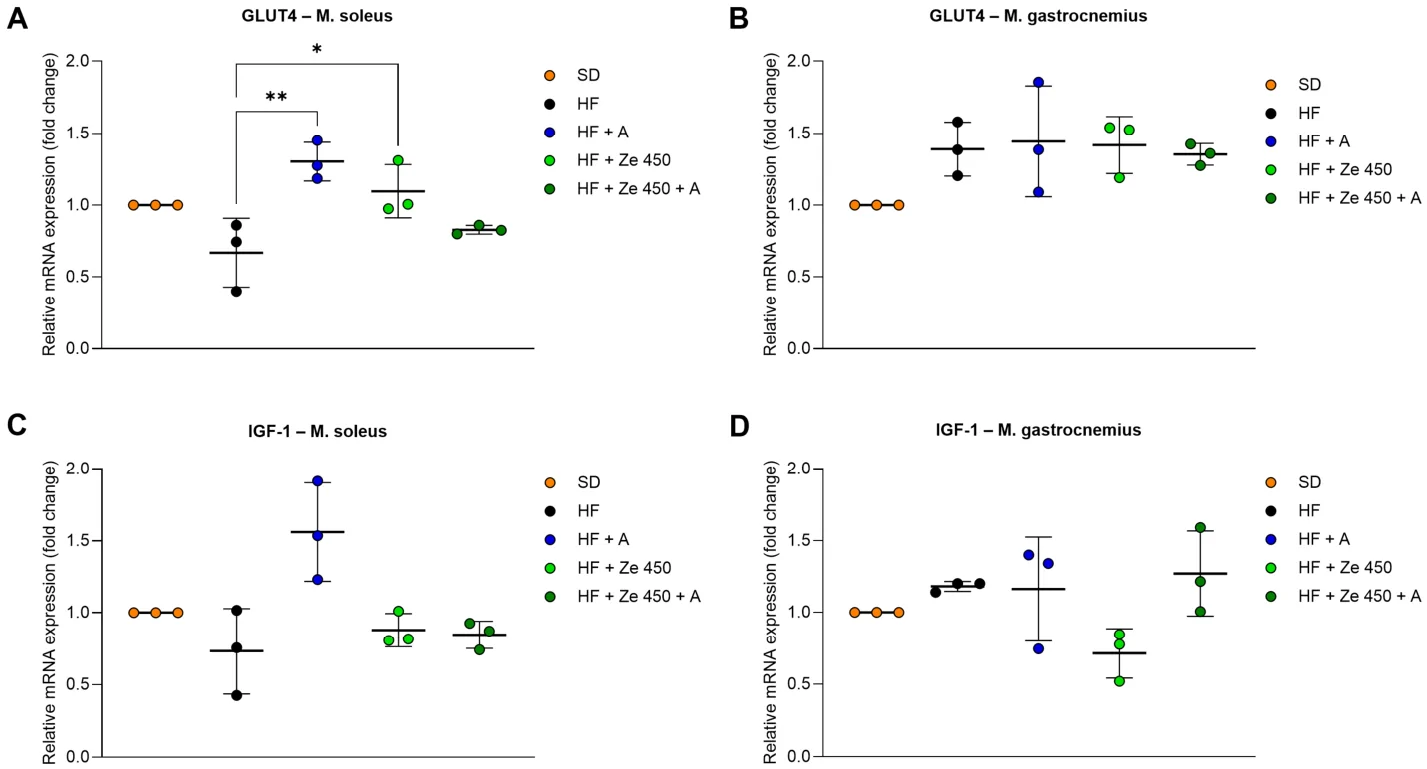
Effects of Ze 450 on glucose transport and myogenic gene expression in skeletal muscle of high-fat diet-fed rats. (**A**,**C**) Relative mRNA expression of *Slc2a4* (GLUT4) and *Igf1* (IGF-1) normalized to *mt-Co1* (COX1) in *M. soleus* and (**B**,**D**) *M. gastrocnemius* from animals fed a standard diet (SD), high-fat diet (HF), HF + A (=voluntary running activity), HF + Ze 450 (130 mg/kg body weight/day), or HF + Ze 450 + A. All data are presented as mean ± standard deviation from *n* = 3 experiments. Samples were pooled from 6 animals per group prior to analysis. Statistical analysis was performed using one-way ANOVA followed by Dunnett’s multiple-comparisons test, comparing SD and treatment groups to the HF control. * *p* < 0.05, ** *p* < 0.01.

As shown in Figure 8A, voluntary running activity and Ze 450 significantly increased glucose transporter type 4 (GLUT4) mRNA expression in the soleus compared with the HF group, whereas their combination had no significant effect. No significant differences in GLUT4 mRNA expression were observed among the groups in the gastrocnemius in comparison with the HF group (Figure 8B). Running activity alone was associated with numerically higher IGF-1 mRNA expression in the soleus compared with the HF group, but this difference did not reach statistical significance (Figure 8C). In the gastrocnemius, IGF-1 mRNA expression was numerically lower in the HF + Ze 450 group than in the HF group, but this difference was not statistically significant (Figure 8D).

## 4. Discussion

Weight loss is an important strategy for improving obesity-related health. Beyond body mass reduction, the preservation of skeletal muscle mass and function has emerged as a critical determinant in evaluating the overall efficacy of weight loss interventions [43,44,45,46]. *Cimicifuga racemosa* extract Ze 450 has been reported to regulate energy metabolism and obesity-related pathways [32,47,48]. Our previous study demonstrated that Ze 450 reduced body weight gain and fat accumulation in female rats fed a high-fat diet [36]. However, whether these effects were accompanied by reductions in skeletal muscle mass remained unclear. In the present study, Ze 450 attenuated DEX-induced atrophy in C2C12 myotubes and reduced body weight gain and fat accumulation in vivo without reducing relative skeletal muscle mass or muscle fiber CSA. Food intake and energy intake in this animal cohort have been reported previously [36]. Although gravimetric food intake was lower in the HF-fed animals than in the SD group, no significant differences in energy intake were observed among the experimental groups. However, food intake was recorded at the cage level rather than individually. Therefore, subtle individual differences in intake or an influence of Ze 450 on diet palatability cannot be excluded. Ze 450 was also associated with muscle-specific differences in markers related to mitochondrial content and lipid and glucose metabolism.

Our in vitro experiments indicate that the effects of Ze 450 on myotube diameter differed from those of DHT. Ze 450 had limited effects on myotube diameter in the absence of DEX but attenuated DEX-induced myotube atrophy. The glutaraldehyde-induced autofluorescence approach enabled morphometric assessment of myotube diameter but did not provide information on sarcomere organization. Thus, the observed changes in myotube diameter provide morphological evidence, while their implications for sarcomeric integrity remain to be determined.

Previous work by Günther et al. [32] reported that Ze 450 did not induce significant cell death in C2C12 myoblasts. In addition, Rabenau et al. [30] reported findings consistent with maintained viability in HT22 neuronal cells exposed to 100 µg/mL Ze 450, a concentration tenfold higher than the highest concentration used in the present study. These findings provide supporting context regarding the cellular tolerability of Ze 450; however, cell viability was not directly assessed in differentiated C2C12 myotubes under the 72 h treatment conditions used in our study.

Dexamethasone is a glucocorticoid that induces muscle atrophy processes, such as reduction in myotube diameter and catabolic responses by suppressing protein synthesis, promoting protein degradation [49,50]. Dihydrotestosterone is an androgen receptor agonist known to promote anabolic responses in skeletal muscle [51]. In C2C12 cells, mTOR serves as a central regulator of anabolic responses, orchestrating cellular growth, protein synthesis, and myogenic differentiation [52]. In contrast to DHT, Ze 450 did not increase mTOR mRNA expression. However, mRNA expression does not provide information on mTOR protein abundance, phosphorylation status, or downstream signaling activity. Nevertheless, our results suggest that Ze 450 significantly attenuates DEX-induced myotube atrophy, similar to other plant-derived compounds including ecdysterone, quercetin, myricanol, rosmarinic acid, and diosgenin [53,54]. The DEX-induced myotube atrophy provides a cell model to evaluate the protective role of Ze 450 under catabolic stress, while the underlying anti-catabolic mechanism remains to be established. This protective effect may involve reduced proteolysis, activation of protein synthesis pathways, or improved mitochondrial function. Potential molecular mediators that warrant direct investigation include AKT/mTOR and FOXO3 signaling, as well as MuRF-1 and atrogin-1 [53].

To further investigate this anti-athropic potential, the effects of Ze 450 on skeletal muscle were evaluated in female Wistar rats. The HF diet significantly increased body weight gain, whereas Ze 450, voluntary running activity, and their combination attenuated this increase. Ze 450, voluntary running activity, and their combination also markedly reduced visceral fat mass compared with the HF group. These body weight and visceral fat mass findings have been reported previously for the same experimental cohort [36] and are included here to contextualize the skeletal muscle outcomes. The HF diet increased the absolute masses of both the soleus and gastrocnemius, although these differences were no longer evident after normalization to body weight. Ze 450 did not significantly affect relative soleus or gastrocnemius mass. Likewise, Ze 450 did not reduce the CSA of either *M. soleus* or *M. gastrocnemius*. Thus, the reductions in body weight gain and visceral fat mass were not accompanied by reductions in relative skeletal muscle mass or muscle fiber CSA, indicating preservation of muscle morphology. Voluntary running activity showed a nonsignificant trend toward greater relative gastrocnemius mass. This observation is consistent with previous evidence that HF diet-induced body weight gain can obscure increases in absolute muscle mass after normalization to body weight, whereas exercise-associated increases may remain detectable [55,56,57].

However, skeletal muscle preservation cannot be fully characterized on the basis of muscle mass and CSA alone. Therefore, additional molecular markers related to mitochondrial content and lipid and glucose metabolism were investigated.

To assess skeletal muscle mitochondrial content, the protein levels of COX IV and CS were examined. COX IV is a component of mitochondrial respiratory chain complex IV, whereas CS is a tricarboxylic acid cycle enzyme localized in the mitochondrial matrix. Both COX IV and CS are commonly used as indicators of mitochondrial density in skeletal muscle [58]. COX IV and CS showed similar expression patterns across treatment groups in both the soleus and gastrocnemius, indicating concordance between the two mitochondrial markers.

In line with previous evidence from obese animal models, our results indicate that the high-fat diet decreased COX IV and CS protein expression in the gastrocnemius [59]. In contrast, no comparable reduction was observed in the soleus, consistent with previous evidence that the high-fat diet affects skeletal muscle mitochondrial adaptations in a muscle-type-dependent manner [60]. Voluntary running activity increased mitochondrial content-related markers in both the soleus and gastrocnemius of high-fat diet-fed rats, which is consistent with previous studies showing that exercise training ameliorates impairments in skeletal muscle mitochondrial function and dynamics in obese mice [61].

It is well established that the soleus and gastrocnemius are characterized by distinct intrinsic metabolic phenotypes, reflecting differences in fiber-type composition, basal oxidative capacity, mitochondrial abundance, and substrate preference [62]. The soleus is predominantly composed of oxidative slow-twitch fibers and exhibits high basal mitochondrial content and oxidative capacity, whereas the gastrocnemius is a mixed muscle with a relatively greater glycolytic component and less reliance on β-oxidation [63,64]. Interestingly, Ze 450 did not induce a uniform mitochondrial response in these two muscles. Rather, it decreased COX IV and CS protein abundance in the soleus, suggesting a possible reduction in reliance on mitochondrial oxidative metabolism, with a potential shift toward glycolytic energy supply. Conversely, in the gastrocnemius, Ze 450 increased these mitochondrial markers, suggesting a potential enhancement of oxidative metabolic capacity. This interpretation is also supported by previous findings by Rabenau et al. [30], who demonstrated that Ze 450 acts as a regulator of energy metabolism by directly targeting mitochondria. In their study, Ze 450 reduced mitochondrial oxygen consumption and promoted a metabolic shift from oxidative phosphorylation toward glycolysis, as reflected by increased PDK1 and HXKII protein levels and enhanced glucose uptake. Similar findings were reported in C2C12 muscle cells, where Ze 450 activated AMPK signaling, reduced mitochondrial respiration, enhanced glucose uptake, and appeared to shift cellular energy metabolism toward greater reliance on glucose utilization [32]. The present study extends this concept to skeletal muscle in vivo and indicates muscle-specific differences in COX IV and CS responses to Ze 450. This muscle-specific response may depend on the distinct intrinsic metabolic phenotypes of the two muscles. Fiber-type composition was not directly assessed in the present study. Therefore, we cannot determine whether the treatment-associated changes in COX IV and CS reflect metabolic adaptations within existing fiber types or changes in fiber-type composition. Future studies incorporating MyHC isoform analysis are required to distinguish between these possibilities.

The exploratory IMTG findings showed a pattern consistent with this muscle-type-specific rebalancing hypothesis. In the soleus, the pooled-sample measurement indicated higher IMTG content with Ze 450 (Figure A1, see Appendix A.1), a pattern consistent with the hypothesis that reduced mitochondrial content may lower fatty acid oxidation, potentially allowing more substrate for IMTG accumulation [65,66].

We also examined the impact of the high-fat diet, Ze 450 treatment, voluntary running activity, and their combination on the expression of genes involved in lipolysis and lipogenesis. These results indicate that Ze 450 treatment affects the expression of these genes in skeletal muscle, but the regulatory pattern is complex.

Interestingly, FAS protein expression was downregulated in *M. gastrocnemius* and *M. soleus* by the HF diet. This finding contrasts with a previous report by Funai et al. [67], in which FAS expression was increased after high-fat diet treatment. This discrepancy may be attributable to differences in the stage of obesity. In the present study, rats were pre-obese based on their body weights, whereas the mice in Funai et al. had already developed HFD-induced obesity and insulin resistance. In our study, the decrease in FAS expression may represent an early adaptive response to lipid oversupply before the development of overt obesity. During this pre-obese stage, higher dietary fat availability may suppress endogenous lipogenesis, thereby potentially limiting lipid synthesis and accumulation in skeletal muscle [67,68].

Adipose triglyceride lipase and hormone-sensitive lipase are well-established key enzymes involved in intracellular lipolysis, whereas sterol regulatory element-binding protein 1c and fatty acid synthase are major regulators of lipogenesis [69,70]. Ze 450 alone significantly reduced HSL mRNA expression in both muscles compared with the HF group, whereas ATGL mRNA expression was only numerically lower. The combination of Ze 450 and voluntary running activity significantly reduced ATGL mRNA expression in the soleus. Voluntary running activity alone significantly reduced HSL mRNA expression in the gastrocnemius. The lower expression of lipolysis-associated genes may be consistent with the exploratory observation of higher IMTG content in the pooled Ze 450 sample. However, whether these transcriptional differences translate into reduced lipolytic activity and IMTG hydrolysis remains to be determined [71,72]. The mRNA expression of lipogenic genes, including SREBP-1c and FAS, showed less consistent patterns and did not fully parallel the corresponding protein-level changes. The discrepancy between FAS mRNA and protein responses may reflect post-transcriptional regulation, as mRNA abundance does not necessarily predict protein abundance [36,73,74]. Similarly, IGF-1 mRNA responses were not accompanied by significant changes in muscle mass or fiber CSA following Ze 450 treatment. Accordingly, the mRNA measurements in the present study should be interpreted as indicators of transcriptional responses rather than as direct measures of functional protein activity. For example, RNA-binding proteins, including HuR and AU-rich element-binding proteins such as AUF1, play important roles in modulating mRNA stability and translation, thereby potentially influencing protein output independently of mRNA abundance [75].

As previously reported for the same animal cohort [36], Ze 450 and voluntary running activity, alone or in combination, showed a nonsignificant trend toward improved glucose tolerance compared with the HF control group. Therefore, we also analyzed GLUT4 mRNA expression across the treatment groups in *M. soleus* and *M. gastrocnemius*. The GLUT4 mRNA expression pattern is consistent with the proposed metabolic rebalancing. Ze 450 increased GLUT4 mRNA expression only in the soleus, which may facilitate glucose uptake and provide more substrate for glycolysis, thereby supporting a shift toward glycolytic energy supply [30]. In contrast, Ze 450 did not increase GLUT4 mRNA levels in the gastrocnemius, supporting the interpretation that the gastrocnemius may not shift toward glycolysis. Nevertheless, as GLUT4 was assessed only at the mRNA level, this result cannot confirm increased glucose uptake, GLUT4 protein abundance, membrane translocation, or glycolytic flux; therefore, this interpretation should be viewed with caution [76]. Future studies should incorporate protein expression, phosphorylation status, enzymatic activity, and subcellular localization analyses, particularly GLUT4 translocation and lipid droplet-associated protein localization [77,78,79,80], to better determine the functional relevance of these molecular changes. In this context, and in line with our previous findings in the same animal cohort, Ze 450 may be discussed within the broader framework of exercise-associated effects [36]. The present muscle-specific responses, together with the previously observed divergence from canonical exercise-associated molecular signaling, suggest that Ze 450 reproduces selected exercise-associated metabolic features through partially distinct mechanisms.

Taken together, our findings suggest that Ze 450 attenuates body weight gain and fat accumulation without concomitant reductions in the measured skeletal muscle morphological parameters under metabolic stress conditions. Together with the in vitro findings under DEX-induced catabolic stress, these results support potential anti-catabolic activity of Ze 450, although the present study design does not establish a direct muscle-protective effect in vivo. Because the animals used in this study were female and represented a pre-obese state rather than established obesity, these findings may be more relevant to populations undergoing early adverse changes in body composition, such as perimenopausal women. However, the present study cannot determine whether the observed responses are sex-specific. Their applicability to males therefore remains to be established. Furthermore, caution is warranted when extrapolating these findings to established obesity or older populations. Given that the menopausal transition is commonly accompanied by increased fat mass, reduced lean mass, and impaired muscle quality [81,82,83], Ze 450 warrants further investigation as a potential strategy to limit body weight gain while maintaining skeletal muscle parameters in perimenopausal populations.

## 5. Conclusions

In C2C12 myotubes, Ze 450 had limited effects on myotube diameter under basal conditions and did not increase mTOR mRNA expression but significantly attenuated DEX-induced myotube atrophy, supporting an anti-atrophic effect under catabolic stress rather than hypertrophic activity. In high-fat diet-fed rats, Ze 450 attenuated body weight gain and reduced visceral fat mass without reductions in either relative skeletal muscle mass or muscle fiber CSA, indicating preservation of muscle morphology alongside the observed changes in body composition. Moreover, Ze 450 was associated with muscle-specific differences in metabolic profiles between the gastrocnemius and soleus muscles, suggesting a potential role as a muscle-type-specific metabolic rebalancer. The underlying mechanisms and functional consequences of these metabolic changes remain to be established. Further mechanistic and functional studies, including direct assessments of pathway activity and muscle function, are required to establish their biological relevance.

## Figures and Tables

**Table 1:** Sequences of the primers used in real-time PCR. For, forward primer; Rev, reverse primer.

Primer		Sequence (5′ → 3′)
IGF-1 (Rat)	For	CGCTGAAGCCTACAAAGTC
	Rev	TGTTTTGCAGGTTGCTCAAG
GLUT4 (Rat)	For	TATTTGGCTTTGTGGCCTTC
	Rev	CGGCAAATAGAAGGAAGACG
SREBP-1C (Rat)	For	GTACCTGCGGGACAGCTTAG
	Rev	CAGGTCATGTTGGAAACCAC
FAS (Rat)	For	GGCTAGAGACCTTGGCACTG
	Rev	TAGCCCTCTGCTCTGGTCAC
ATGL (Rat)	For	CTTTCTCCCAGTGGACCGAG
	Rev	GAGCGGCTTACACACATCCT
HSL (Rat)	For	AAAAGCTGAACCTGGGGGAGG
	Rev	TCGGTCACAGCAGCAGTAGAA
COX1 (Rat)	For	CGTCACAGCCCATGCATTCG
	Rev	CTGTTCATCCTGTTCCAGCTC
GAPDH (C2C12)	For	TGCGACTTCAACAGCAACTC
	Rev	GCCTCTCTCTTGCTCAGTGTCC
IGF-1 (C2C12)	For	TGACATGCCCAAGACTCT
	Rev	TGTGGCATTTCTGCTCCGTGG
mTOR (C2C12)	For	TCCGAGAGATGAGTCAAGAGG
	Rev	CACCTTCCACTCCTATGAGGC

**Figure A2:**
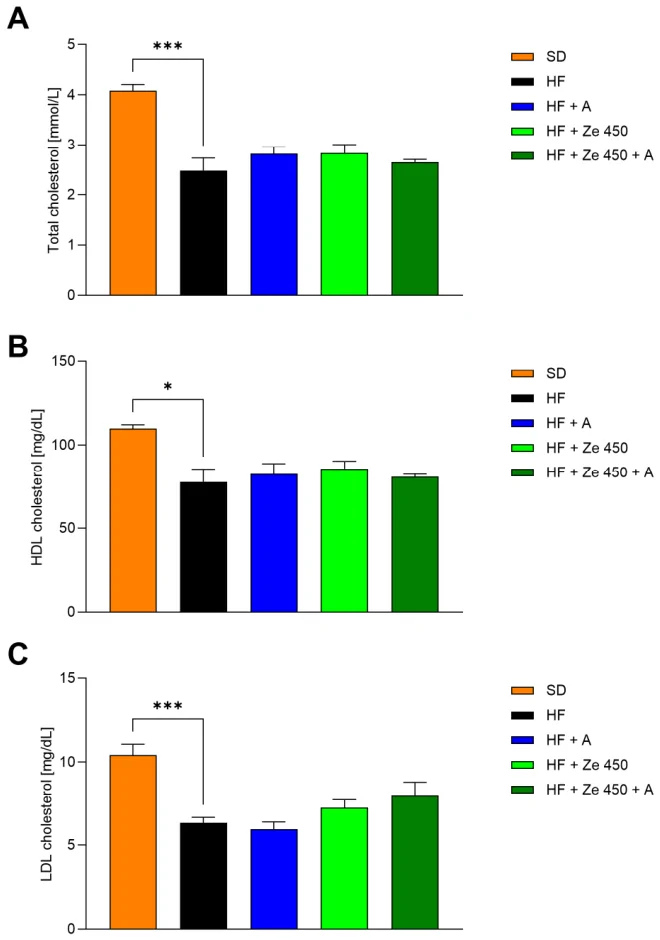
Effects of Ze 450 on serum cholesterol concentrations of high-fat diet-fed rats. (**A**) Serum total cholesterol, (**B**) HDL cholesterol, and (**C**) LDL cholesterol levels from animals fed a standard diet (SD), high-fat diet (HF), HF + A, HF + Ze 450 (130 mg/kg body weight/day), or HF + Ze 450 + A. Serum cholesterol measurements are based on individual animals (*n* = 6 per group). Statistical analysis was performed using one-way ANOVA followed by Dunnett’s multiple-comparisons test, comparing SD and treatment groups to the HF control. * *p* < 0.05, *** *p* < 0.001.

## Data Availability

The datasets presented in this article are not readily available due to time limitations. Requests to access the datasets should be directed to the corresponding author.

## Abbreviations

The following abbreviations are used in this manuscript:

A	voluntary running activity
AKT	protein kinase B
AMPK	AMP-activated protein kinase
ANOVA	analysis of variance
ATGL	adipose triglyceride lipase
AUF1	AU-rich element RNA-binding protein 1
COX IV	cytochrome c oxidase subunit IV
CS	citrate synthase
CSA	cross-sectional area
DEX	dexamethasone
DHT	dihydrotestosterone
DMEM	Dulbecco’s modified Eagle medium
DMSO	dimethyl sulfoxide
DPBS	Dulbecco’s phosphate-buffered saline
FAS	fatty acid synthase
FCS	fetal calf serum
FOXO3	forkhead box O3
GAPDH	glyceraldehyde-3-phosphate dehydrogenase
GLUT4	glucose transporter type 4
H&E	hematoxylin and eosin
HDL	high-density lipoprotein
HF	high-fat diet
HRP	horseradish peroxidase
HRT	hormone replacement therapy
HSL	hormone-sensitive lipase
HuR	human antigen R
HXKII	hexokinase II
IGF-1	insulin-like growth factor 1
IMTG	intramuscular triglyceride
LDL	low-density lipoprotein
mTOR	mechanistic target of rapamycin
MuRF-1	muscle RING finger protein 1
MyHC	Myosin Heavy Chain
OCT	optimal cutting temperature compound
PDK1	pyruvate dehydrogenase kinase 1
PVDF	polyvinylidene fluoride
RT-qPCR	reverse transcription quantitative polymerase chain reaction
SD	standard diet or standard deviation, depending on context
SDS-PAGE	sodium dodecyl sulfate–polyacrylamide gel electrophoresis
SREBP-1c	sterol regulatory element-binding protein 1c
UPLC-MS	ultra-performance liquid chromatography–mass spectrometry

## Appendix A

### Appendix A.1. Exploratory Analysis of Intramuscular Triglyceride (IMTG) Content Following Ze 450 Supplementation of High-Fat Diet-Fed Rats

As shown in Figure A1, IMTG content was lower in the HF group than in the SD group in both *M. soleus* and *M. gastrocnemius*. Activity was associated with a modestly higher IMTG content compared with HF feeding alone. Ze 450 supplementation was also associated with higher IMTG content, with the most pronounced difference observed in *M. soleus*. However, IMTG content was lower in the HF + Ze 450 + A group than in the HF + Ze 450 group in both muscles. Although these exploratory findings are based on single pooled samples and require confirmation using independent biological replicates, they suggest a muscle-specific pattern of IMTG responses to Ze 450 and voluntary activity.

### Appendix A.2. Effects of Ze 450 on Serum Cholesterol Concentrations of High-Fat Diet-Fed Rats

HF feeding significantly reduced serum total cholesterol, high-density lipoprotein (HDL) cholesterol, and low-density lipoprotein (LDL) cholesterol levels compared with SD. Other treatments did not lead to further significant changes in cholesterol levels relative to HF. The total cholesterol results have previously been reported using the same cohort of animals [36].

## References

[1] Cruz-Jentoft A.J., Bahat G., Bauer J., Boirie Y., Bruyère O., Cederholm T., Cooper C., Landi F., Rolland Y., Sayer A.A. (2019). Sarcopenia: Revised European consensus on definition and diagnosis. Age Ageing.

[2] Cruz-Jentoft A.J., Sayer A.A. (2019). Sarcopenia. Lancet.

[3] Prado C.M., Phillips S.M., Gonzalez M.C., Heymsfield S.B. (2024). Muscle matters: The effects of medically induced weight loss on skeletal muscle. Lancet Diabetes Endocrinol..

[4] Neeland I.J., Linge J., Birkenfeld A.L. (2024). Changes in lean body mass with glucagon-like peptide-1-based therapies and mitigation strategies. Diabetes Obes. Metab..

[5] Locatelli J.C., Costa J.G., Haynes A., Naylor L.H., Fegan P.G., Yeap B.B., Green D.J. (2024). Incretin-Based Weight Loss Pharmacotherapy: Can Resistance Exercise Optimize Changes in Body Composition?. Diabetes Care.

[6] Cava E., Yeat N.C., Mittendorfer B. (2017). Preserving Healthy Muscle during Weight Loss. Adv. Nutr..

[7] Lowrie F.J., Rahimi M.M., Hogan C.M., Tran D.L., Marshall N.S., Phillips C.L., Gordon C.J., Yee B.J., Machan E.A. (2025). The impact of exercise on fat free mass reduction during very low energy diet-induced weight loss in adults with overweight or obesity: A systematic review and meta-analysis. Obes. Res. Clin. Pract..

[8] Weiss E.P., Jordan R.C., Frese E.M., Albert S.G., Villareal D.T. (2017). Effects of weight loss on lean mass, strength, bone, and aerobic capacity. Med. Sci. Sports Exerc..

[9] Juppi H.-K., Sipilä S., Cronin N.J., Karvinen S., Karppinen J.E., Tammelin T.H., Aukee P., Kovanen V., Kujala U.M., Laakkonen E.K. (2020). Role of Menopausal Transition and Physical Activity in Loss of Lean and Muscle Mass: A Follow-Up Study in Middle-Aged Finnish Women. J. Clin. Med..

[10] Smith-Ryan A.E., Hirsch K.R., Cabre H.E., Gould L.M., Gordon A.N., Ferrando A.A. (2023). Menopause Transition: A Cross-Sectional Evaluation on Muscle Size and Quality. Med. Sci. Sports Exerc..

[11] Bondarev D., Finni T., Kokko K., Kujala U.M., Aukee P., Kovanen V., Laakkonen E.K., Sipilä S. (2021). Physical Performance During the Menopausal Transition and the Role of Physical Activity. J. Gerontol. Ser. A Biol. Sci. Med. Sci..

[12] Menzies C., Bowtell R., Shur N., Brook M.S. (2026). Menopause, Female Sex Hormones, Skeletal Muscle Mass and Muscle Protein Turnover in Humans. J. Cachexia Sarcopenia Muscle.

[13] Chaston T.B., Dixon J.B., O’Brien P.E. (2007). Changes in fat-free mass during significant weight loss: A systematic review. Int. J. Obes..

[14] Stiegler P., Cunliffe A. (2006). The role of diet and exercise for the maintenance of fat-free mass and resting metabolic rate during weight loss. Sports Med..

[15] Sousa-Victor P., Gutarra S., García-Prat L., Rodriguez-Ubreva J., Ortet L., Ruiz-Bonilla V., Jardí M., Ballestar E., González S., Serrano A.L. (2014). Geriatric muscle stem cells switch reversible quiescence into senescence. Nature.

[16] Kitajima Y., Ono Y. (2016). Estrogens maintain skeletal muscle and satellite cell functions. J. Endocrinol..

[17] Collins B.C., Arpke R.W., Larson A.A., Baumann C.W., Xie N., Cabelka C.A., Nash N.L., Juppi H.-K., Laakkonen E.K., Sipilä S. (2019). Estrogen Regulates the Satellite Cell Compartment in Females. Cell Rep..

[18] Larson A.A., Shams A.S., McMillin S.L., Sullivan B.P., Vue C., Roloff Z.A., Batchelor E., Kyba M., Lowe D.A. (2022). Estradiol deficiency reduces the satellite cell pool by impairing cell cycle progression. Am. J. Physiol. Cell Physiol..

[19] Österdahl M.F., Ni Lochlainn M., Welch C., Rymer J., Ashworth M., Whitney J., Duncan E.L., Steves C.J. (2025). Systematic review on the relationship between menopausal hormone replacement therapy, sarcopenia, and sarcopenia-related parameters. Maturitas.

[20] Greising S.M., Baltgalvis K.A., Lowe D.A., Warren G.L. (2009). Hormone therapy and skeletal muscle strength: A meta-analysis. J. Gerontol. Ser. A Biol. Sci. Med. Sci..

[21] Dam T.V., Dalgaard L.B., Ringgaard S., Johansen F.T., Bisgaard Bengtsen M., Mose M., Lauritsen K.M., Ørtenblad N., Gravholt C.H., Hansen M. (2020). Transdermal Estrogen Therapy Improves Gains in Skeletal Muscle Mass After 12 Weeks of Resistance Training in Early Postmenopausal Women. Front. Physiol..

[22] Zhang G.-Q., Chen J.-L., Luo Y., Mathur M.B., Anagnostis P., Nurmatov U., Talibov M., Zhang J., Hawrylowicz C.M., Lumsden M.A. (2021). Menopausal hormone therapy and women’s health: An umbrella review. PLoS Med..

[23] Schellenberg R., Saller R., Hess L., Melzer J., Zimmermann C., Drewe J., Zahner C. (2012). Dose-Dependent Effects of the *Cimicifuga racemosa* Extract Ze 450 in the Treatment of Climacteric Complaints: A Randomized, Placebo-Controlled Study. Evid. Based Complement. Altern. Med. eCAM.

[24] Wuttke W., Seidlová-Wuttke D. (2015). Black cohosh (*Cimicifuga racemosa*) is a non-estrogenic alternative to hormone replacement therapy. Clin. Phytosci..

[25] Castelo-Branco C., Gambacciani M., Cano A., Minkin M.J., Rachoń D., Ruan X., Beer A.-M., Schnitker J., Henneicke-von Zepelin H.-H., Pickartz S. (2021). Review & meta-analysis: Isopropanolic black cohosh extract iCR for menopausal symptoms—An update on the evidence. Climacteric J. Int. Menopause Soc..

[26] EMA/HMPC (2018). European Union Herbal Monograph on Cimicifuga racemosa (L.) Nutt., Rhizoma: Draft-Revision.

[27] EMA/HMPC (2018). Assessment Report on Cimicifuga racemosa (L.) Nutt., Rhizoma—Revision 1, EMA/HMPC/48744/2017.

[28] Cătăuță L.M., Senn R., Schenk A., Chaanin A., Halbsguth C., Danton O. (2026). Bridging Pharmacopeial Standards and Chemical Reality of Triterpene Glycosides in *Actaea racemosa*. Plants.

[29] Garita-Hernandez M., Calzado M.A., Caballero F.J., Macho A., Muñoz E., Meier B., Brattström A., Fiebich B.L., Appel K. (2006). The growth inhibitory activity of the *Cimicifuga racemosa* extract Ze 450 is mediated through estrogen and progesterone receptors-independent pathways. Planta Medica.

[30] Rabenau M., Dillberger B., Günther M., Krippner S., Butterweck V., Boonen G., Drewe J., Eckert G.P., Culmsee C. (2021). *Cimicifuga racemosa* Extract Ze 450 Re-Balances Energy Metabolism and Promotes Longevity. Antioxidants.

[31] Rabenau M., Unger M., Drewe J., Culmsee C. (2019). Metabolic switch induced by *Cimicifuga racemosa* extract prevents mitochondrial damage and oxidative cell death. Phytomed. Int. J. Phytother. Phytopharm..

[32] Günther M., Schnierle P., Rose T., Schlegel J., Boonen G., Drewe J., Muñoz E., Fiebich B.L., Culmsee C. (2024). AMPK Activation by *Cimicifuga racemosa* Extract Ze 450 Is Associated with Metabolic Effects and Cellular Resilience against Age-Related Pathologies in Different Tissue Cell Types. Pharmaceutics.

[33] Moser C., Vickers S.P., Brammer R., Cheetham S.C., Drewe J. (2014). Antidiabetic effects of the *Cimicifuga racemosa* extract Ze 450 in vitro and in vivo in ob/ob mice. Phytomed. Int. J. Phytother. Phytopharm..

[34] Friederichsen L., Nebel S., Zahner C., Bütikofer L., Stute P. (2020). Effect of *CIMicifuga racemosa* on metaBOLIC parameters in women with menopausal symptoms: A retrospective observational study (CIMBOLIC). Arch. Gynecol. Obstet..

[35] Habersatter E., Kostov T., Laing N., Drewe J., Boonen G., Butterweck V., Diel P.R. (2026). Pilot Investigation on the Metabolic Effects of *Cimicifuga racemosa* Extract Ze 450 and Voluntary Physical Activity in Female Rats. Int. J. Mol. Sci..

[36] Habersatter E., Chen S., Kostov T., Drewe J., Boonen G., Butterweck V., Diel P. (2026). Exercise-associated and metabolic effects of *Cimicifuga racemosa* extract Ze 450 in a high-fat diet model in female rats. Biomed. Pharmacother..

[37] Zheng W., Hemker M.L., Xie M., Soukup S.T., Diel P. (2018). Anabolic activity of a soy extract and three major isoflavones in C2C12 myotubes. Planta Medica.

[38] Weigt C., Hertrampf T., Zoth N., Fritzemeier K.H., Diel P. (2012). Impact of estradiol, ER subtype specific agonists and genistein on energy homeostasis in a rat model of nutrition induced obesity. Mol. Cell. Endocrinol..

[39] Ceglia L., Niramitmahapanya S., Price L.L., Harris S.S., Fielding R.A., Dawson-Hughes B. (2013). An evaluation of the reliability of muscle fiber cross-sectional area and fiber number measurements in rat skeletal muscle. Biol. Proced. Online.

[40] Weigt C., Hertrampf T., Kluxen F.M., Flenker U., Hülsemann F., Fritzemeier K.H., Diel P. (2013). Molecular effects of ER alpha-and beta-selective agonists on regulation of energy homeostasis in obese female Wistar rats. Mol. Cell. Endocrinol..

[41] Kostov T., Diel P., Isenmann E. (2024). Examination of the anabolic activity and mechanisms of action of the combination of diosgenin and ecdysterone in C2C12 myotubes. Toxicol. Lett..

[42] Timón-Gómez A., Nývltová E., Abriata L.A., Vila A.J., Hosler J., Barrientos A. (2018). Mitochondrial cytochrome c oxidase biogenesis: Recent developments. Seminars in Cell & Developmental Biology.

[43] McCarthy D., Berg A. (2021). Weight Loss Strategies and the Risk of Skeletal Muscle Mass Loss. Nutrients.

[44] Magkos F., Fraterrigo G., Yoshino J., Luecking C., Kirbach K., Kelly S.C., de Las Fuentes L., He S., Okunade A.L., Patterson B.W. (2016). Effects of moderate and subsequent progressive weight loss on metabolic function and adipose tissue biology in humans with obesity. Cell Metab..

[45] Ryan D., Yockey S. (2017). Weight loss and improvement in comorbidity: Differences at 5%, 10%, 15%, and over. Curr. Obes. Rep..

[46] Harper M.-E., Dent R.R., McPherson R. (2025). High-quality weight loss in obesity: Importance of skeletal muscle. Diabetes.

[47] Rachoń D., Vortherms T., Seidlová-Wuttke D., Wuttke W. (2008). Effects of black cohosh extract on body weight gain, intra-abdominal fat accumulation, plasma lipids and glucose tolerance in ovariectomized Sprague–Dawley rats. Maturitas.

[48] Sun Y., Yu Q., Shen Q., Bai W., Kang J. (2016). Black Cohosh Ameliorates Metabolic Disorders in Female Ovariectomized Rats. Rejuvenation Res..

[49] Dubashynskaya N.V., Bokatyi A.N., Skorik Y.A. (2021). Dexamethasone conjugates: Synthetic approaches and medical prospects. Biomedicines.

[50] Menconi M., Gonnella P., Petkova V., Lecker S., Hasselgren P.-O. (2008). Dexamethasone and corticosterone induce similar, but not identical, muscle wasting responses in cultured L6 and C2C12 myotubes. J. Cell. Biochem..

[51] Dubois V., Laurent M., Boonen S., Vanderschueren D., Claessens F. (2012). Androgens and skeletal muscle: Cellular and molecular action mechanisms underlying the anabolic actions. Cell. Mol. Life Sci..

[52] Atherton P.J., Smith K., Etheridge T., Rankin D., Rennie M.J. (2010). Distinct anabolic signalling responses to amino acids in C2C12 skeletal muscle cells. Amino Acids.

[53] Dai W., Wu J., Ma X., Wu M., Li K., Gao H., Wang W., Xiao W. (2026). Plant-derived natural products alleviate dexamethasone-induced skeletal muscle atrophy by modulating FoxO. Int. J. Mol. Med..

[54] Parr M.K., Zhao P., Haupt O., Ngueu S.T., Hengevoss J., Fritzemeier K.H., Piechotta M., Schlörer N., Muhn P., Zheng W.Y. (2014). Estrogen receptor beta is involved in skeletal muscle hypertrophy induced by the phytoecdysteroid ecdysterone. Mol. Nutr. Food Res..

[55] Kim Y.J., Kim H.J., Lee W.J., Seong J.K. (2020). A comparison of the metabolic effects of treadmill and wheel running exercise in mouse model. Lab. Anim. Res..

[56] Kanazawa Y., Ikeda-Matsuo Y., Sato H., Nagano M., Koinuma S., Takahashi T., Suzuki H., Miyachi R., Shigeyoshi Y. (2023). Effects of obesity in old age on the basement membrane of skeletal muscle in mice. Int. J. Mol. Sci..

[57] Messa G.A., Piasecki M., Hurst J., Hill C., Tallis J., Degens H. (2020). The impact of a high-fat diet in mice is dependent on duration and age, and differs between muscles. J. Exp. Biol..

[58] Koshinaka K., Honda A., Masuda H., Sato A. (2020). Effect of quercetin treatment on mitochondrial biogenesis and exercise-induced AMP-activated protein kinase activation in rat skeletal muscle. Nutrients.

[59] Jun L., Knight E., Broderick T.L., Al-Nakkash L., Tobin B., Geetha T., Babu J.R. (2024). Moderate-intensity exercise enhances mitochondrial biogenesis markers in the skeletal muscle of a mouse model affected by diet-induced obesity. Nutrients.

[60] Gómez-Pérez Y., Capllonch-Amer G., Gianotti M., Lladó I., Proenza A.M. (2012). Long-term high-fat-diet feeding induces skeletal muscle mitochondrial biogenesis in rats in a sex-dependent and muscle-type specific manner. Nutr. Metab..

[61] Heo J.W., No M.H., Cho J., Choi Y., Cho E.J., Park D.H., Kim T.W., Kim C.J., Seo D.Y., Han J. (2021). Moderate aerobic exercise training ameliorates impairment of mitochondrial function and dynamics in skeletal muscle of high-fat diet-induced obese mice. FASEB J..

[62] Schiaffino S., Reggiani C. (2011). Fiber types in mammalian skeletal muscles. Physiol. Rev..

[63] Dong H., Tsai S.-Y. (2023). Mitochondrial properties in skeletal muscle fiber. Cells.

[64] Picard M., Hepple R.T., Burelle Y. (2012). Mitochondrial functional specialization in glycolytic and oxidative muscle fibers: Tailoring the organelle for optimal function. Am. J. Physiol. Cell Physiol..

[65] Corcoran M.P., Lamon-Fava S., Fielding R.A. (2007). Skeletal muscle lipid deposition and insulin resistance: Effect of dietary fatty acids and exercise. Am. J. Clin. Nutr..

[66] Guo Z. (2007). Intramyocellular lipid kinetics and insulin resistance. Lipids Health Dis..

[67] Funai K., Song H., Yin L., Lodhi I.J., Wei X., Yoshino J., Coleman T., Semenkovich C.F. (2013). Muscle lipogenesis balances insulin sensitivity and strength through calcium signaling. J. Clin. Investig..

[68] Duarte J.A., Carvalho F., Pearson M., Horton J.D., Browning J.D., Jones J.G., Burgess S.C. (2014). A high-fat diet suppresses de novo lipogenesis and desaturation but not elongation and triglyceride synthesis in mice. J. Lipid Res..

[69] Zimmermann R., Strauss J.G., Haemmerle G., Schoiswohl G., Birner-Gruenberger R., Riederer M., Lass A., Neuberger G., Eisenhaber F., Hermetter A. (2004). Fat mobilization in adipose tissue is promoted by adipose triglyceride lipase. Science.

[70] Griffin M.J., Sul H.S. (2004). Insulin regulation of fatty acid synthase gene transcription: Roles of USF and SREBP-1c. IUBMB Life.

[71] Alsted T.J., Nybo L., Schweiger M., Fledelius C., Jacobsen P., Zimmermann R., Zechner R., Kiens B. (2009). Adipose triglyceride lipase in human skeletal muscle is upregulated by exercise training. Am. J. Physiol. Endocrinol. Metab..

[72] Kraemer F.B., Shen W.-J. (2002). Hormone-sensitive lipase. J. Lipid Res..

[73] Franks A., Airoldi E., Slavov N. (2017). Post-transcriptional regulation across human tissues. PLoS Comput. Biol..

[74] Esau C., Davis S., Murray S.F., Yu X.X., Pandey S.K., Pear M., Watts L., Booten S.L., Graham M., McKay R. (2006). miR-122 regulation of lipid metabolism revealed by in vivo antisense targeting. Cell Metab..

[75] Brennan C., Steitz J. (2001). HuR and mRNA stability. Cell. Mol. Life Sci. CMLS.

[76] Hager S.R., Pastorek D., Jochen A.L., Meier D. (1991). Divergence between GLUT4 mRNA and protein abundance in skeletal muscle of insulin resistant rats. Biochem. Biophys. Res. Commun..

[77] Richter E.A., Hargreaves M. (2013). Exercise, GLUT4, and skeletal muscle glucose uptake. Physiol. Rev..

[78] Huang S., Czech M.P. (2007). The GLUT4 glucose transporter. Cell Metab..

[79] Sakamoto K., Holman G.D. (2008). Emerging role for AS160/TBC1D4 and TBC1D1 in the regulation of GLUT4 traffic. Am. J. Physiol. Endocrinol. Metab..

[80] Mason R.R., Meex R.C., Russell A.P., Canny B.J., Watt M.J. (2014). Cellular localization and associations of the major lipolytic proteins in human skeletal muscle at rest and during exercise. PLoS ONE.

[81] McGregor R.A., Cameron-Smith D., Poppitt S.D. (2014). It is not just muscle mass: A review of muscle quality, composition and metabolism during ageing as determinants of muscle function and mobility in later life. Longev. Heal..

[82] Larsson L., Degens H., Li M., Salviati L., Lee Y.I., Thompson W., Kirkland J.L., Sandri M. (2019). Sarcopenia: Aging-related loss of muscle mass and function. Physiol. Rev..

[83] Greendale G.A., Sternfeld B., Huang M., Han W., Karvonen-Gutierrez C., Ruppert K., Cauley J.A., Finkelstein J.S., Jiang S.-F., Karlamangla A.S. (2019). Changes in body composition and weight during the menopause transition. JCI Insight.

